# Ultraconserved long non-coding RNA uc.63 in breast cancer

**DOI:** 10.18632/oncotarget.10572

**Published:** 2016-07-13

**Authors:** Alberto Marini, Anna Maria Lena, Emanuele Panatta, Cristina Ivan, Leng Han, Han Liang, Margherita Annicchiarico-Petruzzelli, Nicola Di Daniele, George A. Calin, Eleonora Candi, Gerry Melino

**Affiliations:** ^1^ Medical Research Council, Toxicology Unit, Hodgkin Building, University of Leicester, Leicester, UK; ^2^ Department of Experimental Medicine and Surgery, University of Rome “Tor Vergata”, Rome, Italy; ^3^ Department of Experimental Therapeutics and The Center for RNA interference and non-coding RNA, The University of Texas M.D. Anderson Cancer Center, Houston, TX, USA; ^4^ Department of Biochemistry and Molecular Biology, The University of Texas Health Science Center at Houston McGovern Medical School, Houston, TX, USA; ^5^ Department of Bioinformatics and Computational Biology, The University of Texas M.D. Anderson Cancer Center, Houston, TX, USA; ^6^ IDI-IRCCS, Biochemistry Laboratory, Rome, Italy

**Keywords:** lncRNA, T-UCRs, breast cancer, apoptosis, prognosis

## Abstract

Transcribed-ultraconserved regions (T-UCRs) are long non-coding RNAs (lncRNA) encoded by a subset of long ultraconserved stretches in the human genome. Recent studies revealed that the expression of several T-UCRs is altered in cancer and growing evidences underline the importance of T-UCRs in oncogenesis, offering also potential new strategies for diagnosis and prognosis. We found that overexpression of one specific T-UCRs named uc.63 is associated with bad outcome in luminal A subtype of breast cancer patients. uc.63 is localized in the third intron of exportin-1 gene (*XPO1*) and is transcribed in the same orientation of its host gene. Interestingly, silencing of uc.63 induces apoptosis *in vitro*. However, silencing of host gene *XPO1* does not cause the same effect suggesting that the transcription of uc.63 is independent of *XPO1*. Our results reveal an important role of uc.63 in promoting breast cancer cells survival and offer the prospect to identify a signature associated with poor prognosis.

## INTRODUCTION

Breast cancer is the most common tumor and the second leading cause of cancer deaths in women with over 1,400,000 new cases diagnosed every year worldwide. From the clinical point of view, breast cancer is characterized by wide heterogeneity. Gene expression profiling, as well as immuno-histochemical analysis of estrogen receptor (ER) α, progesterone receptor (PR) and human epidermal growth factor receptor 2 (HER2) can classify human breast cancers in four major histopatological/therapeutical subtypes: luminal A, luminal B, HER2 and basal-like [1, 2]. Each category differs in terms of prognosis and response to therapy. The ER positive group (luminal A and B) is amenable to hormone therapy, with several genomic tests to support in predicting outcomes [3]. HER2 group includes patients responsive to trastuzumab therapy, which has led to a great clinical success [3]. Basal cancers, also known as triple-negative breast cancers, are characterized by the lack of expression ER, PR and HER2 [3]. This phenotype makes basal tumors difficult to treat, more aggressive and with poor prognosis compared to the other subtypes [4].

During the last few years, new evidences estimated that approximately 95% of the human genome transcripts are non-coding RNAs (ncRNAs) [5, 6, 7]. Long non-coding RNAs (lncRNAs) are a subtype of ncRNAs molecules longer than 200nt involved in several biological and pathological processes, such as differentiation [8], immune response [9], metabolism [10] and cancer development and progression [11, 12, 13]. Interestingly, many cancer specific lncRNAs have been identified as biomarkers for metastasis or prognosis, for example metastasis associated long antisense transcript-1 (MALAT-1) in lung adenocarcinoma [14], HOX transcript antisense RNA (HOTAIR) in pancreatic and breast cancer [15, 11] and colon cancer associated transcripts (CCAT2) [16].

Recently, a new class of lncRNAs has been characterized, called transcribed-ultraconserved regions (T-UCRs). T-UCRs are encoded by a subset of ultraconserved regions (UCRs) in the DNA, which are absolutely conserved between orthologous loci of the human, rat, and mouse genomes [17, 18]. Interestingly, the expression of several T-UCRs is altered in tumorigenesis [18, 22] and it occurs in different ways. Frequently, fragile sites and cancer-associated genomic regions (CAGRs) contain T-UCRs, thus affecting their transcription when rearranged [18]. Furthermore, interactions with miRNAs have been reported to regulate T-UCRs levels in chronic lymphocytic leukemia (CLL) [18] and in neuroblastoma [19], whereas in prostate cancer and in other cancer cell lines (breast, colorectal, lung, lymphoma and leukemia) methylation of CpG islands in the promoter has been found involved in T-UCRs silencing [20, 21]. Finally, Ferdin et al. [23] discovered that a subset of T-UCRs (uc.63, uc.73, uc.106, uc.134 and uc.475) is regulated by hypoxia, a typical feature of tumor aggressiveness, and showed that uc.475 has a key role in supporting cancer cell proliferation in low-oxygen conditions [23]. Taken together, all these evidences underline the importance of T-UCRs in cancer biology and suggest the relevance of T-UCRs-expression profiles as useful tools to differentiate human cancer types and correlate with diagnosis and prognosis.

Here, we characterized a specific hypoxia-induced T-UCRs, named uc.63, in breast cancer. The data available on this T-UCRs reveal high levels of the transcript in colorectal cancer but there are no evidences about its biological role and prognostic significance in tumors. We found that uc.63 overexpression is correlated with poor prognosis in a luminal A subgroup of breast cancer patients and we discovered a key role in controlling cancer cells survival.

## RESULTS

### Long non-coding RNA uc.63 overexpression is associated with poor prognosis in breast cancer

**Figure 1:**
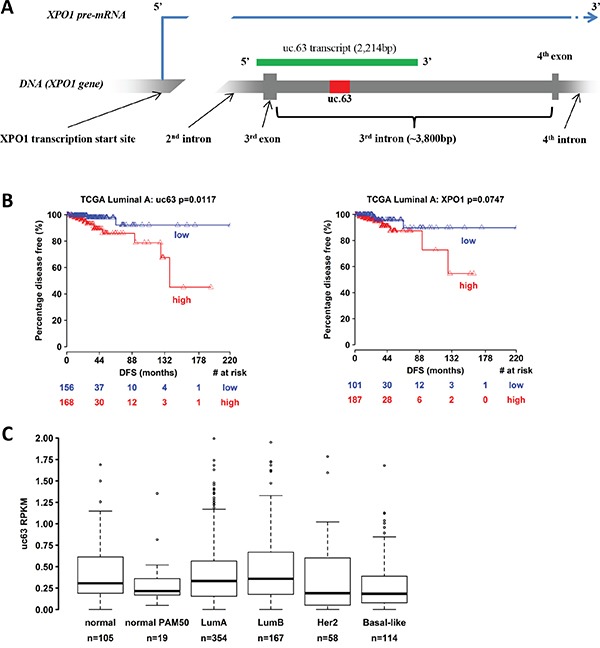
Bionformatic analysis of uc.63 expression in breast cancer patients **A**. Scheme of uc.63 transcript and locus in chromosome 2 (grey: *XPO1* gene / red: uc.63 ultraconserved sequence / green: uc.63 transcript). **B**. Disease-free survival information of breast cancer patients were downloaded from TCGA portal (http://tcga-data.nci.nih.gov/) [3, 87]. Kaplan-Meier method was used to generate percentage disease-free curves. **C**. Expression analysis of uc.63 transcript in PAM50 breast cancer subgroups. PAM50 gene signature relies on 50 discriminatory genes to segregate tumors into luminal A, luminal B, HER2–enriched and basal-like [88]. Samples labelled with “normal PAM50” are tumor subtype with no corresponding clinical-pathologic category (normal-like). Samples categorized as “normal” are normal breast tissue.

uc.63 ultraconserved region (278bp) is localized on the chromosome 2p15 (chr2:61752501-61752778, GRCh37/hg19), in the third intron of *XPO1* gene (Exportin-1, CRM1) (Figure 1A). The sequence is conserved in a broad spectrum of species and in mouse is localized in the fourth intron of *Xpo1* orthologous gene (Supplementary Table S1)

In MCF-7 breast cancer cell line, the transcription of this region leads to a longer transcript, approximately of 2,200nt [23], thus overlapping the third exon of *XPO1* gene (Figure 1A). Furthermore, cellular fractionation experiment revealed a predominant nuclear localization of uc.63 [23]. Overexpression of uc.63 transcript has been found in patients with colorectal cancer and its upregulation is induced by hypoxic conditions [23] *in vitro*. Despite these data, the clinical relevance of uc.63 is still unknown.

In order to investigate the prognostic significance of uc.63 overexpression in breast cancer, we performed a bionformatic analysis by using TCGA (The Cancer Genome Atlas) portal [3, 87], which contains expression profiles data of over 2,000 breast cancer specimens. We found that the increased expression of uc.63 is associated with reduced disease-free survival in luminal A subtype of breast cancer patients (Figure 1B, left panel). Interestingly, XPO1 overexpression is not correlated with the free survival, thus demonstrating that high levels of XPO1 are not linked to relapse in this group of tumors (Figure 1B, right panel). Despite these data, analysis of uc.63 expression in breast cancer subgroups of PAM50 gene signature does not reveal any significant upregulation of uc.63 transcript both in luminal A and in all of other subtypes (Figure 1C), suggesting that the prognostic value of uc.63 is limited to a cohort of patients with more aggressive luminal A tumors which develop a relapse of the disease.

Taken together, these data show a correlation between uc.63 upregulation and aggressiveness in luminal A breast cancers, identifying a potentially useful molecular signature for the prognosis of this neoplasia.

### Analysis of uc.63 transcript

**Figure 2:**
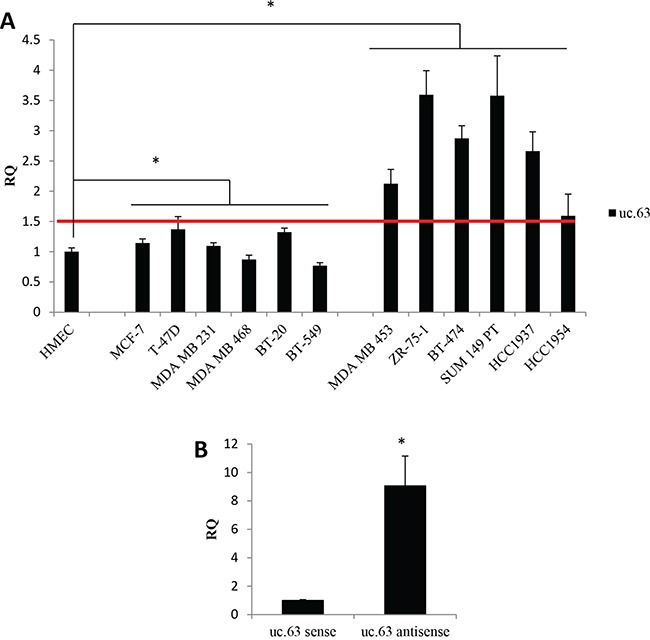
uc.63 expression in human breast cancer lines **A**. Expression analysis of uc.63 in breast cancer cell lines. 500,000 cells were plated in complete medium and uc.63 level was evaluated by RT-qPCR after 48h. HMECs were used as reference sample and TBP was used as endogenous control. Median of all RQ values in tumor cells (RQ=1.50) is represented by a red line. Cell lines with low (left) and high (right) uc.63 levels were separated. *p < 0.01 vs HMEC. **B**. Strand-specific RT-qPCR. Total RNA isolated from MDA MB 453 cells was used to perform RT-qPCR with strand-specific primers complementary to uc.63 antisense or uc.63 sense transcripts. TBP was used as endogenous control. uc.63 sense was used as reference sample. *p < 0.01 vs uc.63 sense.

In order to analyze *in vitro* the uc.63 transcript, we designed specific primers targeting uc.63 ultraconserved sequence. First, we evaluated uc.63 expression in a panel of breast cancer cell lines by RT-qPCR, using as a reference sample HMEC cells (Human Mammary Epithelial Cells), which resemble the normal breast epithelium (Figure 2A). The analyzed cell lines showed high variability in terms of uc.63 expression. For the sake of simplicity, we discriminated cell lines with low (MCF-7, T-47D, MDA MB 231, MDA MB 468, BT-20, BT-549) and high (MDA MB 453, ZR-75-1, BT-474, SUM 149 PT, HCC1937, HCC1954) uc.63 levels by calculating the median of all RQ values in tumor cells (Figure 2A). For following analysis, we used total RNA from MDA MB 453 breast cancer cell line derived from metastatic breast carcinoma and showing high expression of uc.63 (Figure 2A).

Given that uc.63 is localized inside *XPO1* gene, we asked whether uc.63 is transcribed in the same orientation of host gene in these cells. To address this point, we carried out a strand-specific RT-qPCR making two reverse transcription reaction mix, each one containing the primer complementary to the different forms of the transcript. We found that antisense transcript is the predominant form of uc.63 expressed in MDA MB 453, being sense transcript poorly detectable (Figure 2B, Supplementary Figure S1A). As shown in Supplementary Figure S1B, uc.63 antisense has the same sequence of *XPO1* primary transcript, completely matching the region between the end of the second intron and a part of the third intron and containing the third exon (Figure 1A). Thus, we clearly demonstrated that uc.63 RNA is transcribed in the same orientation of *XPO1* mRNA. Our results are in agreement with previously published data [23], which reported the same transcription orientation in MCF-7 breast cancer cell line.

Although uc.63 promoter has not been characterized yet, these data demonstrate that uc.63 transcript has the same orientation of host gene's mRNA. Furthermore, the presence in the putative uc.63 promoter region at 5′ of *XPO1* gene [23] of hypoxia-inducible factor (HIF) candidate binding sites, which may drive uc.63 expression in hypoxic conditions, further confirm the same transcription orientation.

### Biological effects of uc.63

**Figure 3:**
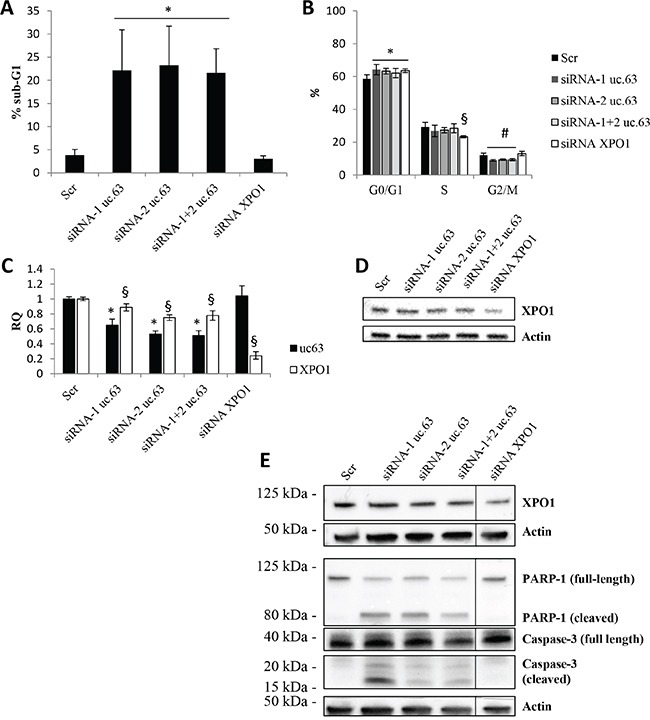
uc.63 knockdown induces apoptosis in MDA MB 453 cell line **A**. sub-G1 events were quantified by using flow cytometry analysis after propidium iodide staining of MDA MB 453 cell line. 500,000 cells were plated and then silenced with siRNA (20nM) targeting uc.63 or XPO1 for 48h. *p < 0.01 vs Scr. **B**. Cell cycle analysis following uc.63 or XPO1 knockdown. Analysis was carried out by using ModFit software. *p < 0.05 vs G0/G1 Scr; §p < 0.01 vs S Scr; #p < 0.05 vs G2/M Scr. **C**. RT-qPCR for uc.63 and XPO1 after uc.63 or XPO1 knockdown. TBP was used as endogenous control. *p < 0.01 vs Scr, §p < 0.01 vs Scr. **D**. XPO1 immunoblot analysis following uc.63 or XPO1 knockdown. β-actin was used as endogenous control. **E**. Immunoblot analysis of apoptotic markers PARP-1 and caspase-3. β-actin was used as endogenous control.

Given that there are no evidences about the biological role of uc.63 overexpression in breast cancer, we used high-expressing uc.63 MDA MB 453 cell line and a siRNA-based approach by designing two different specific siRNAs targeting uc.63 ultraconserved sequence, in order to investigate the phenotypic effect of uc.63 knockdown (KD). We found that uc.63 downregulation leads to cell death in MDA MB 453 cells (Figure 3A, 3C), associated with an increase in G0/G1 cells and a reduction in G2/M events (Figure 3B). Of note, the same results were obtained by using uc.63 siRNAs alone or in combination. uc.63 siRNAs also cause a slight but significant decrease in *XPO1* mRNA level, probably by targeting the primary transcript (the third intron) of host gene (Figure 3C). However, uc.63 siRNAs do not affect XPO1 protein level (Figure 3D, Supplementary Figure S2), thus excluding the possibility that XPO1 downregulation leads to the phenotypic effect. Finally, we asked whether apoptosis is involved in inducing cell death of MDA MB 453 cells *in vitro*. Thus, we analyzed the activation status of apoptotic markers PARP-1 and Caspase-3. Western blot analysis revealed both PARP-1 and Caspase-3 activated forms (cleaved) after uc.63 KD, thus confirming our hypothesis (Figure 3E). Taken together, these findings show that uc.63 plays an important role in controlling survival in breast cancer cells.

### *XPO1* mRNA and uc.63 are two independent transcripts

In order to further demonstrate that XPO1 is not involved in the biological effects induced by uc.63 downregulation, we carried out XPO1 KD by using a pool of four specific siRNAs. As expected, XPO1 downregulation does not affect survival of MDA MB 453 cells (Figure 3A, 3C, 3D) and we only detected a reduction in S phase associated with increased G1/G0 events (Figure 3B). In agreement, we did not find any activation of apoptotic pathway, as indicated by PARP-1 and Caspase-3 immunoblot analysis (Figure 3E). Altogether, this functional analysis strongly suggests that uc.63 and *XPO1* mRNA are two independent transcripts and only uc.63 is able to modulate apoptosis.

## DISCUSSION

Breast cancer is a heterogeneous disease with different histopathological, genetic and genomic variations, and clinical outcomes, which make difficult to define therapies and prognostic factors. Genetic and epigenetic changes, as well as aberrant interactions with tumor microenvironment, drive a multistep process characterized by progressive deregulation of proliferation, survival, differentiation and metabolism, finally giving rise to aggressive and metastatic breast tumor [26, 27]. Despite novel therapies, ~30% of treated patients later relapse and over 450,000 die yearly [25]. For all these reasons, gene-expression profiles of breast carcinomas are useful tools in order to identify prognostic markers and predict response to treatments.

A relevant player in breast cancer, as well as in cancer in general, is the entire p53 family. The p53 gene family of transcription factors includes three very similar genes that codify for the p53, p73 and p63 proteins [28, 29, 30, 31]. p53 shows a very complex transcription activation program ranging from metabolism [32, 33, 34, 35], mitochondria and ROS regulation [36, 37], DNA damage repair [38, 39, 40, 41], autophagy [42, 43], stemness and lineage determination [44, 45]. At the molecular level, this complexity can be studied from different aspects; indeed, detailed analysis are under way on its splicing isoforms [46, 47], its connection and regulation to miRNAs [48, 49, 50, 51] as well as its stability and degradation [24, 52, 53, 54, 55, 56, 57, 58]. Furthermore, in keeping with the progress in understanding p53 function, there is also a strong effort in investigating innovative therapeutic cues [59, 60, 61, 62, 63]. Of note, the other members of the family, p63 and p73, which were discovered only ten years ago, have an evident complex interplay with p53 itself [64, 65, 66, 67, 68]. In the case of p63, its function is crucial for skin development and homeostasis, as well as for cancer [69, 70, 66, 71, 72, 73, 74], whereas p73 is a critical player in brain development and homeostasis, as well as in tumors [75, 33, 76, 77, 78, 79].

In the last few years, ncRNAs are emerging as new actors in breast cancer biology [80]. In this regard, miRNAs play a key role in proliferation, metastasis and drug resistance in breast tumors [81]. For example, miR-34 family members, which are directly regulated by p53, control cell cycle and apoptosis by modulating the levels of proteins such as CDK4, CDK6, cyclin D1, cyclin E2 and BCL-2 [81]. Furthermore, miR-125a and miR-125b are able to suppress HER2 expression, thus decreasing both growth and invasiveness [82], while miR-200 family members are directly involved in regulating epithelial-mesenchymal transition [83]. Lastly, miR-221 and miR-222 contribute to tamoxifen resistance, downregulating ER-α level [84]. Interestingly, among ncRNAs, lncRNAs have recently appeared as relevant drivers of breast cancer. The long non-coding transcript HOTAIR has been described to remodel chromatin through polycomb repressive complex 2 (PRC2), thus increasing cancer invasiveness *in vitro* and *in vivo*, and its overexpression is an independent predictor of overall survival and progression-free survival [11]. On the other hand, GAS5 lncRNA acts as an oncosuppressor, being downregulated in breast tumors and leading to apoptosis and block of proliferation *in vitro* [85]. Despite these data, the new class of lncRNAs T-UCRs is not characterized in breast cancer and our knowledge about their role in breast tumor biology is very limited.

Dysregulation of T-UCRs expression is emerging as a common feature of human cancers. Here, we discovered that aberrant expression of uc.63 in breast cancer cells is important in controlling survival. In fact, uc.63 KD leads to apoptosis *in vitro* and this effect is specific for the T-UCR. Although uc.63 is localized inside the *XPO1* gene and is transcribed in the same transcriptional orientation, exportin-1 KD does not recapitulate the phenotypic effect of uc.63 downregulation, suggesting that uc.63 and *XPO1* mRNA are two independent transcripts with two independent promoters. However, even though our functional analysis strongly suggests an independent transcription, further studies will be needed in order to better characterize uc.63 RNA and its regulation. Of note, hypoxia has been already demonstrated to transcriptionally induce uc.63 [23] and this feature strongly supports the link between cancer aggressiveness and its expression.

As for uc.73 in colorectal cancer cells [18], we found that uc.63 is able to modulate apoptosis in breast cancer cells. This data suggests that uc.63 acts as an oncogene and its overexpression is important in promoting cell survival and tumor growth. At the moment, we have no evidences about the proper mechanism by which uc.63 controls survival in tumor cells and further analysis are necessary to deeply understand the cellular pathways involved in this process.

Several evidences demonstrated that, compared to normal counterpart, neoplastic cells show a unique expression profile of T-UCRs, suggesting a significant role of T-UCRs in the malignant process [18]. Variations in ultraconserved non-coding transcript levels offer a new strategy for tumor diagnosis and prognosis. In our study we found that, in the specific context of luminal A breast cancer tumors, uc.63 overexpression is associated with reduced disease-free survival, thus correlating this T-UCR with aggressiveness. Of note, XPO1 overexpression is not correlated with relapse in luminal A subtype, suggesting that exportin-1 does not affect disease-free survival in this particular group of tumors. Moreover, this data further confirms the independence in terms of expression between XPO1 and uc.63. Therefore, we propose uc.63 expression as a potential tool useful for prognosis in breast cancer patients

In summary, our study revealed an important role of uc.63 in breast cancer and could potentially offer the perspective of identification of T-UCRs' signatures associated with prognosis of the disease.

## MATERIALS AND METHODS

### Cell cultures and growth conditions

MDA MB 453, MDA MB 231, MDA MB 468, BT-20, BT-549, BT-474, MCF-7 and T-47D cells were cultured in Dulbecco's Modified Eagle's Medium DMEM (LONZA) supplemented with 10% FBS (Gibco) and Penicillin-Streptomycin (Gibco), respectively 100 U/mL and 100 μg/mL. SUM 149 PT were cultured in HAMs F12 medium supplemented with 5% FBS, 2mM glutamine (Gibco), 5μg/mL insulin, 10mM HEPES, 1μg/mL hydrocortisone and Penicillin-Streptomycin. HCC 1937 and HCC 1954 were cultured in RPMI medium supplemented with 10% FBS, 2mM glutamine and Penicillin-Streptomycin. ZR-75-1 were cultured in RPMI medium supplemented with 10% FBS, 1mM sodium pyruvate, 10mM HEPES, 2mM glutamine and Penicillin-Streptomycin. HMEC were obtained from Gibco and were cultured in HuMEC medium consists of HuMEC Basal Serum-Free Medium (Gibco, cat. 12753018) supplemented with HuMEC Supplement Kit (Gibco, cat. 12755013). The HuMEC Supplement Kit includes 5mL of a supplement mix containing epidermal growth factor, hydrocortisone, isoproterenol, transferrin, and insulin, and 25mg of bovine pituitary extract.

### siRNA transfection

MDA MB 453 were transfected using Lipofectamine RNAiMAX transfection reagent (Invitrogen) by following manufacturer's protocol. 500,000 cells were plated one day before transfection. uc.63 and XPO1 were silenced using 20nM siRNA for 48h. uc.63 siRNAs target the following sequences: uc.63 siRNA-1, GACATTACTAATGTTTAAGTTGA; uc.63 siRNA-2, TTCTTCAATTTACATAAATTACA. Both siRNAs were provided by Qiagen. XPO1 knockdown was performed by using a pool of four different siRNA (Gene Solution siRNA XPO1, Qiagen, cat. 1027416 - 2991182).

### Primers

uc.63 ultraconserved sequence was downloaded from Bejerano et al. [17]. This sequence was used both for primers and siRNAs design. For the sake of simplicity, we used the complementary sequence of uc.63: uc.63 forward primer, CAGTGTTTGCCTGTTTGCTTGC; uc.63 reverse primer, CCTGTTGCTTTCTTTCTGTTCCTC. XPO1 mRNA was detected using the following primer: XPO1 forward primer, CTCATTGTTTCCCAGCATTCCTTG; XPO1 reverse primer, CCCGTATCTGCGACATTCCTCATAG. TATA-box binding protein (TBP) was used as reference gene in all RT-qPCR analysis: TBP forward primer, TCAAACCCAGAATTGTTCTCCTTAT; TBP reverse primer, CCTGAATCCCTTTAGAATAGGGTAGA.

### RNA extraction and reverse transcription (RT)

Total RNA was extracted from cultured cells using RNAeasy Mini Kit by following manufacturer's protocol (Qiagen). RNA concentrations were measured with spectrophotometer NanoDrop ND-1000 instrument (NanoDrop Tachnologies, Termo Scientific). Reverse transcription of RNA to total cDNA was performed on 1μg of RNA using GoTaq Reverse Transcription System (Promega) by following manufacturer's protocol, in a final reaction of 40μl (1X Reaction Buffer 5X, 5mM MgCl_2_, 0.5mM dNTPs, 1μg random primers, 320U reverse transcriptase, 20U ribonuclease inhibitor).

Strand specific RT was made in 20μL where 100ng of RNA were transcribed to strand specific cDNA (1X Reaction Buffer 5X, 5mM MgCl_2_, 0.5mM dNTPs, 0.5μM strand-specific primers, 160U reverse transcriptase, 20U ribonuclease inhibitor). Two reverse transcription reaction mix were made for strand-specific cDNA synthesis: one to detect uc.63 sense oriented transcript (using forward primer for uc.63 and reverse primer for TBP) and one for uc.63 antisense oriented transcript (using reverse primers both for uc.63 and TBP). Of note, we used only TBP reverse primer in both reaction mix because it can recognize TBP's mRNA. Before use in qPCR analysis, strand-specific cDNAs were diluted 5-times with nuclease-free water.

### Quantitative polymerase chain reaction (qPCR)

Real time PCR was performed using GoTaq qPCR Master Mix (Promega) by following manufacturer's protocol, in a final volume of 25μL (1X qPCR Master Mix 2X, 0.4μM forward and reverse primer mix, 1mM MgCl_2_). 2^−ΔΔCt^ method was used for relative quantifications. TBP was used as reference gene in all reactions.

### Western blot

Cells lysis was performed in RIPA buffer (1% NP-40, 0.1% SDS, 150mM NaCl, 50mM Tris-HCl pH=7.5, 0.5% sodium deoxycholate) supplemented with protease inhibitors and 1mM dithiothreitol. Samples were homogenized by vortexing, kept on ice for 20 minutes and then clarified by centrifugation at 13,000rpm for 10 minutes at 4°C. 20-30μg of total proteins were separated by SDS-PAGE and then transferred onto PVDF membrane (GE Healthcare). Immunoblotting was performed using standard protocols with the following primary antibodies: rabbit anti-XPO1 1:200 (Santa Cruz Biotech., cat. sc-5595), mouse anti-PARP-1 (C-2-10) 1:1000 (Enzo Lifescience, cat. BML-SA250), rabbit anti-Caspase-3 (8G10) 1:500 (Cell Signaling, cat. 9665), mouse anti-actin 1:50,000 (Sigma Aldrich, cat. A5441). Caspase-3 antibody detects endogenous levels of full-length (35KDa) and large fragment (17/19 KDa) of caspase-3 resulting from cleavage at aspartic acid 175. PARP-1 antibody detects intact PARP (~116KDa) and apoptosis-induced cleavage fragment (~80KDa).

### Bionformatic analysis

We downloaded RNA-seq BAM files from UCSC Cancer Genomics Hub (CGHub,https://cghub.ucsc.edu/) for Breast invasive carcinoma (BRCA), respectively normal cases from TCGA (The Cancer Genome Atlas). TCGA BAM files were generated based on Mapsplice2 algorithm for alignment against the hg19 reference genome using default parameters. We quantified the expression of uc.63 as RPKM (reads per kilobase per million mapped reads) by extracting the number of reads overlapped with uc.63 and normalized by the total mappable reads of each sample as previously described [86]. We downloaded RNASeq (RPKM) Level3 data publicly available from the TCGA (http://tcga-data.nci.nih.gov/) for XPO1 in patients with BRCA. Patient survival information was retrieved from cbioPortal (http://www.cbioportal.org/). Patient PAM50 classification results were retrieved from the Associated Data Files of the Cancer Genome Atlas Research Network [87]. Data are available per request. Analyses were carried out in R statistical environment (version 3.0.1) (http://www.r-project.org/). All tests were two-sided and considered statistical significant at the 0.05 level. We performed Cox regression analysis for associations between survival and uc.63 and XPO1 levels. For the Lumina A subgroup, the disease-free survival analysis yielded for uc.63 a hazard ratio of 3.49 (CI(95%)=(1. 17, 10.34), Wald test p-value =0.0243). We than used the log-rank test to find the point (cut-off) with the most significant (lowest p-value) split in high vs low uc.63 mRNA level groups. The Kaplan-Meier plots were generated for this cut-off (0.48). The numbers of patients at risk in low and high uc.63 groups at different time points are presented at the bottom of the graph. The same analysis revealed that XPO1 expression does not associate with survival in this group (hazard ratio= 1.58, CI(95%)=(0.45, 5.9), Wald test p-value = 0.475). We used same methods as for uc63 to generate Kaplan-Meier plots for XPO1 (cut-off=0.35). The Shapiro-Wilk test was applied and verified that uc.63 expression does not follow a normal distribution in each PAM50 subtype, and normal group. Accordingly, the nonparametric Kruskal-Wallis test was applied to assess the relationship between uc.63 expression and subtype. A box-and-whisker plot (Box plot represents first (lower bound) and third (upper bound) quartiles, whiskers represent 1.5 times the interquartile range) was used to visualize the data (log2(x+1)).

### Flow cytometry assay

Propidium iodide (PI) staining of fixed cells was used to evaluate apoptosis and to analyze the distribution in the different cell cycle phases of the cell population. Cells in G1/G0 phases have a diploid DNA content, whereas cells in G2/M phases have a double DNA content. Cells in S phase have an intermediate DNA content. Apoptotic cells are detectable as G1 sub-population (sub-G1) with hypo-diploid DNA content caused by DNA fragmentation. Briefly, cells were harvested along with their medium, centrifuged at 1,200rpm for 10 minutes and washed in ice-cold PBS. Cells were centrifuged again at 1,200rpm for 10 minutes and then fixed in 500μL methanol-acetone 4:1 at 4°C for 30 minutes. After fixing, 2mL of PBS was added to cells. The suspension was centrifuged at 1,200rpm for 10 minutes and the pellet was then incubated in 50μL of 10kU/mL RNAse (Sigma Aldrich) at room temperature for 15 minutes. Finally, 200μL of 60μg/mL PI (Sigma Aldrich) was added to fixed cells, incubating the suspension for 20 minutes. Analysis of samples was carried out acquiring 10,000-15,000 events/sample on FACS Calibur (BD). ModFit software was used to analyze cell cycle phases.

### Statistical analysis

Data are presented as means ± SD. Statistical analysis of the data was performed by Student's t-test. p-values of ≤ 0.05 were considered statistically significant.

## SUPPLEMENTARY FIGURES AND TABLE



## Abbreviations

T-UCRs: transcribed-ultraconserved regions
lncRNAs: long non-coding RNAs
*XPO1*: exportin-1
TCGA: The Cancer Genome Atlas
HMEC: human mammary epithelial cells
PARP-1: Poly [ADP-ribose] polymerase 1
siRNA: small interfering RNA
