# Supplementary material for: Ultraconserved long non-coding RNA uc.63 in breast cancer
Source: Oncotarget. 2016 Jul 13;8(22):35669–80. doi: 10.18632/oncotarget.10572 (PMC5482607; doi:10.18632/oncotarget.10572)
Supplement: Supplementary file 1 [file oncotarget-08-35669-s001.pdf]

Ultraconserved long non-coding RNA uc.63 in breast cancer

SUPPLEMENTARY FIGURES AND TABLE

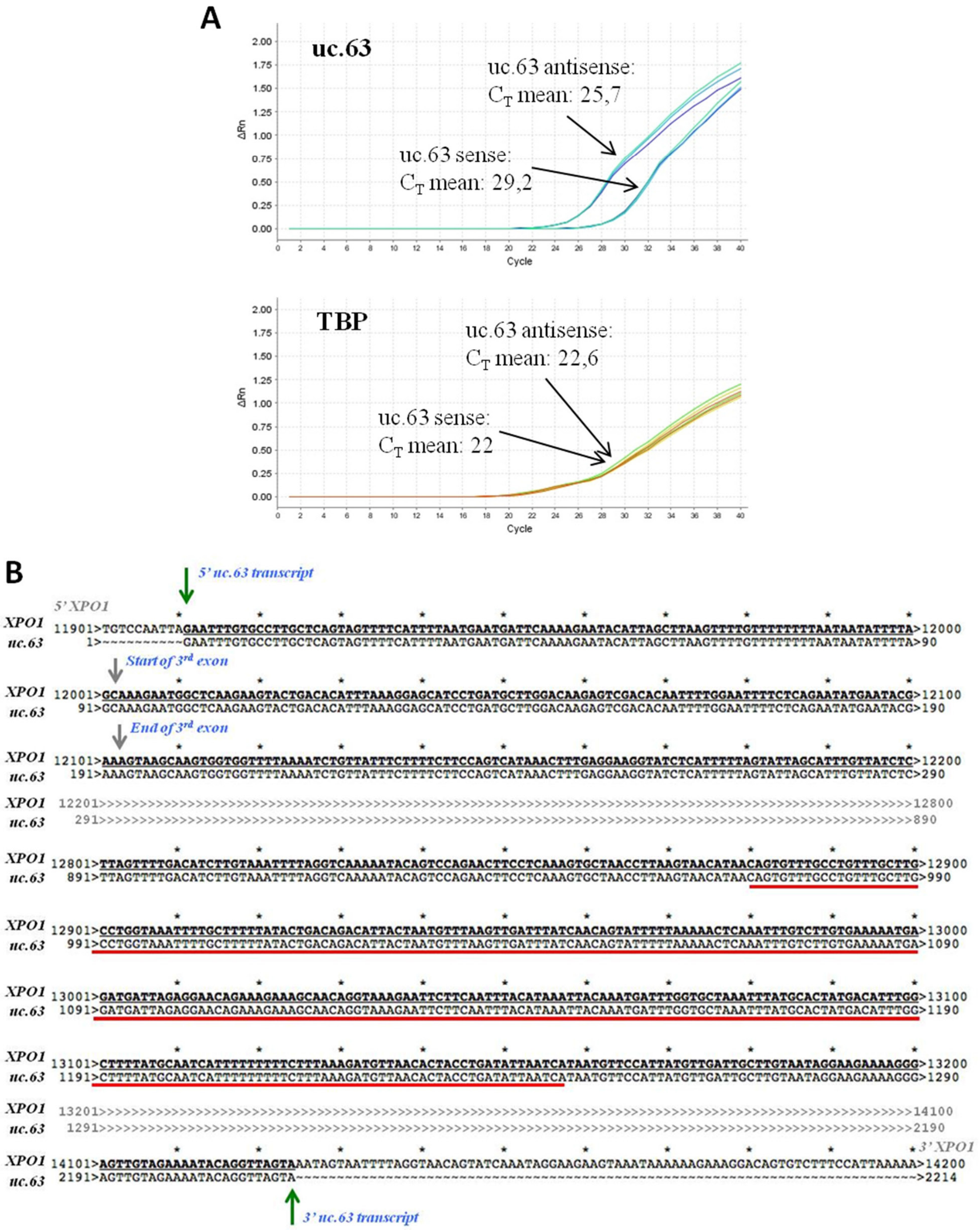

Supplementary Figure S1: uc.63 is transcribed in antisense orientation in MDA MB 453 cells. A. Representative amplification plots of strand-specific RT-qPCR. 500,000 cells were plated in complete medium and RNA was extracted after 48h. Total RNA were used to perform RT-qPCR with strand-specific primers complementary to uc.63 antisense or uc.63 sense transcripts. TBP was used as endogenous control. **B.** Alignment of uc.63 antisense transcript with XPO1 gene (primary transcript). Ultraconserved sequence is underlined in red.

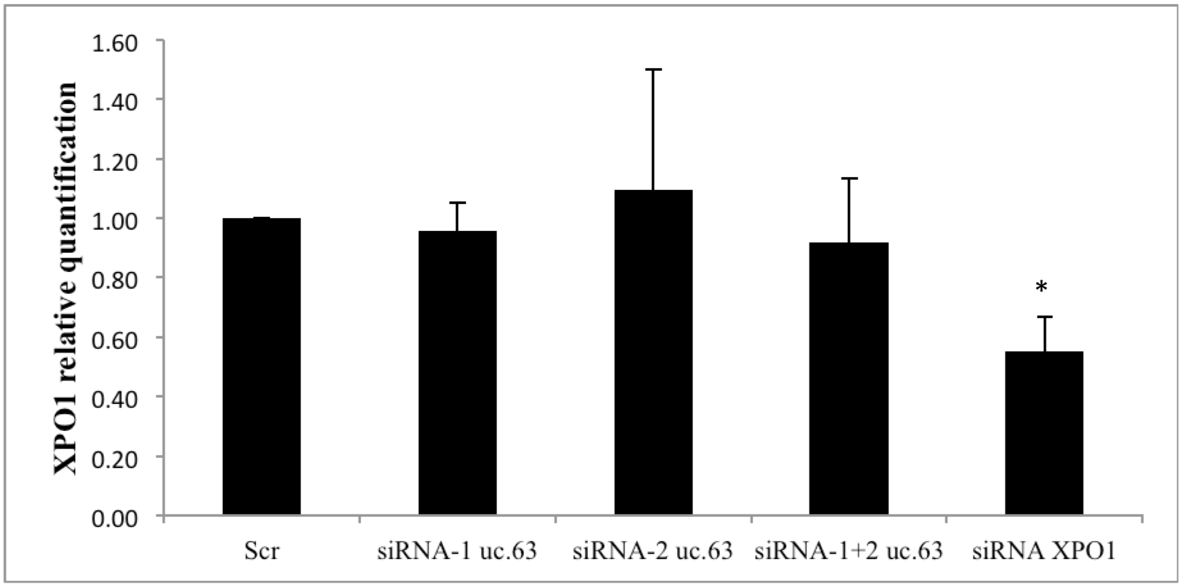

Supplementary Figure S2: XPO1 relative quantification. XPO1 protein levels were quantified using ImageJ software. Scr was used as reference sample and β-actin was used as endogenous control. *$p < 0.01$ vs Scr.

Supplementary Table S1: uc.63 sequence is conserved between different species

Species (assembly)	Region	Identity
Human (GRCh37/hg19)	chr2:61752333-61752781	100%
Mouse (mm10)	chr11:23268537-23268985	99.55%
Chicken (galGal3)	chr3:2356392-2356843	> 95%
Armadillo (dasNov1)	scaffold_24876:11891-12338	99.55%
Opossum (monDom5)	chr1:630963670-630964120	98.23%
Platypus (ornAna1)	Ultra56:4300962-4301415	97.80%

Localization of uc.63 conserved sequence in different species. Information were obtained from UCNEbase tool (http://ccg.vital-it.ch/UCNEbase/), which use a longer version of the conserved sequence (449bp) compared to the one (278bp) reported by Bejerano et al. [17].
